# Safety of Citrate Anticoagulation in CKRT: Monocentric Experience of a Dynamic Protocol of Calcium Monitoring

**DOI:** 10.3390/jcm12165201

**Published:** 2023-08-10

**Authors:** Federico Nalesso, Elisabetta Bettin, Marco Bogo, Martina Cacciapuoti, Leda Cattarin, Giuseppe Scaparrotta, Lorenzo A. Calò

**Affiliations:** Department of Medicine, Nephrology, Dialysis and Transplant, University of Padua, 35128 Padua, Italyrenzcalo@unipd.it (L.A.C.)

**Keywords:** regional citrate anticoagulation, CKRT, AKI, critical Ill patient, blood calcium, anticoagulation protocol

## Abstract

Regional Citrate Anticoagulation (RCA) is considered the first-line anticoagulation for Continuous Kidney Replacement Therapy (CKRT). The RCA requires strict protocols and trained staff to avoid unsafe use and ensure its benefit. We have analyzed all our CKRT prescriptions from December 2020 to April 2022 anonymously, collecting data on CKRT, lab tests, clinical conditions, and complications of RCA. In addition, in order to better detect citrate accumulation, we have performed an RCA protocol by reducing the Ca_Tot_/Ca^2+^ ratio cut-off from 2.50 to 2.40 and increasing the number of calcium checks according to its trend. Among the 374 patients in CKRT, 104 received RCA prescriptions, of which 11 (10.6%) were discontinued: 4 for the suspicion of citrate accumulation, 1 for the development of metabolic alkalosis, 1 for the shift to a different CKRT procedure due to the need for a higher bicarbonate dose, 4 for the elevation of hepatocytolysis indexes, and 1 due to a preemptive discontinuation following massive post-surgery bleeding. None of the patients have had citrate toxicity as indicated by a Ca_Tot_/Ca^2+^ greater than 2.50, and our protocol has allowed the early identification of patients who might develop clinical citrate toxicity.

## 1. Introduction

Patients affected by Acute Kidney Injury (AKI) often require Continuous Kidney Replacement Therapy (CKRT), in particular when hemodynamics are unstable or if fluid balance requires continuous ultrafiltration over 24 h. To maintain CKRT circuit patency, anticoagulation is required, and heparin has been the standard choice for anticoagulation [1]. However, fearing bleeding complications [2], heparin is often administered at subtherapeutic doses, making anticoagulation insufficient and responsible for a poor filter life [3].

Regional Citrate Anticoagulation (RCA) is now the recommended anticoagulation choice for CKRT in patients without contraindications, which, compared with heparin, increases filter life [4] and reduces the rate of bleeding complications, therapy interruptions, and costs [5,6,7,8,9,10,11]. Its use requires accurate protocols and specific training for both medical and nursing staff in order to avoid an unsafe use of RCA and ensure its advantages [12].

Citrate (C_6_H_5_O_7_) is an organic acid commonly used as an anticoagulant in its trisodium citrate form; the anticoagulant properties are related to its high affinity for the divalent calcium ion (Ca^2+^). When citrate is added to the blood, it binds calcium in the citrate-calcium complexes (CCC), decreasing the level of ionized free calcium below 0.35 mmol/L and blocking coagulation [13,14].

The protocols for RCA management differ by citrate solution (citrate concentration in the pre-dilution reinfusion fluid) and CKRT techniques (Continuous Veno-Venous Hemofiltration, CVVH; Continuous Veno-Venous Hemodialysis, CVVHD; Continuous Veno-Venous Hemodiafiltration, CVVHDF).

Common elements of all CKRT techniques in RCA are: (a) the pre-filter citrate solution administration at the required dose to reach approximately 3 to 4 mmol of citrate per liter of blood treated in the circuit in order to decrease ionized calcium to the target range of 0.2–0.35 mmol/L; and (b) the calcium chloride infusion directly through a separated central line to compensate for calcium loss in the effluent fluid in the form of CCC. Citrate and calcium infusions are adjusted according to the ionized calcium post-filter and patient ionized calcium, respectively.

The most feared and potentially lethal complication of RCA is citrate accumulation. In the absence of citrate blood level monitoring and a specific checking protocol for the patient’s total and ionized calcium, the diagnosis of citrate accumulation remains relatively complex [15]. On the contrary, when a strict protocol for the patient’s safety is followed, this complication is rare [16].

The citrate accumulation should be distinguished from other conditions resulting in acid-base abnormalities during RCA, such as citrate net overload (alkalosis) or insufficient citrate delivery (acidosis).

It is well known that the patient’s capacity to metabolize citrate is saturable, and if citrate administration exceeds this capacity, the CCCs remain in the blood and cannot be identified without a routine assay of citrate blood level. In clinical practice, RCA treatments are performed without evaluation of CCCs; thus, citrate accumulation can only be suspected via its indirect signs [17]. The most reliable sign for citrate accumulation is an increased Ca_Tot_/Ca^2+^ ratio that demonstrates an increase in the anion-bound serum calcium level [18]. The CaTot/Ca^2+^ ratio has a significant clinical use, as a value higher than 2.5 is indicative of citrate accumulation, and the trend toward this value is highly indicative of ongoing citrate accumulation [19].

In addition to the CaTot/Ca^2+^ ratio, other signs are commonly observed during citrate accumulation that should not be considered diagnostic criteria but represent warning signs of potential citrate accumulation. The increase in calcium substitutions might suggest the absence of calcium release from CCC, which if leads to hypocalcemia, may cause severe complications.

Another indirect sign of citrate accumulation is the recurrence of high anion gap metabolic acidosis and increased serum lactate levels as an expression of the primary process impairing the tricarboxylic acid cycle, reducing citrate metabolism and limiting pyruvate metabolism, leading to lactate generation.

During CKRT-RCA, net citrate overload is a common complication that is, however, easy to manage. In this situation, the patient’s capacity to metabolize citrate is not reached, and all CCCs are metabolized; ionized calcium levels remain normal, and no increase in the Ca_Tot_/Ca^2+^ ratio is observed. The metabolism of citrate and the concomitant load of sodium ions lead to plasma alkalinization. In the case of insufficient citrate delivery, the patient presents an insufficient alkalotic load, resulting in residual metabolic acidosis with a normal Ca_Tot_/Ca^2+^ ratio and calcium substitution rate.

Nowadays, the monitors for CKRT are easy to use in the CKRT-RCA procedure [20], although in cases of incorrect circuit setup, excess citrate infusion might occur, as in the administration of citrate in post-dilution instead of pre-dilution.

Citrate administration is always coupled with the blood pump, avoiding the accidental infusion of citrate when the blood flow is stopped due to an alarm.

The citrate removal at the filter can be impaired, resulting in excess citrate delivery to the patient, such as during CVVH with a too low ultrafiltration rate or CVVHD with an insufficient dialysate rate. If these treatments are prescribed incorrectly [16], the patient may be exposed to a higher clinical risk of citrate accumulation, especially during the sudden worsening of the patient’s clinical conditions. The strict use of a protocol for the prescription would therefore be able to avoid this complication.

**Figure 1:**
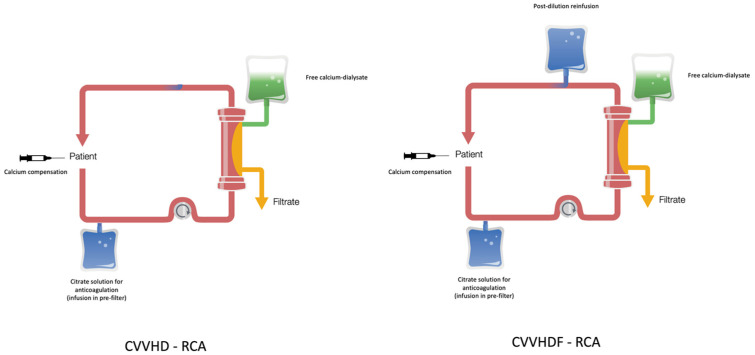
CVVHD and CVVHDF in regional citrate anticoagulation (RCA).

CVVHDF-RCA is the most common technique in RCA, while CVVHD-RCA is the simplest of these techniques (Figure 1).

In critically ill patients with AKI requiring CKRT, citrate metabolism can be reduced. The capacity to metabolize citrate is, in fact, a dynamic process depending on hemodynamic conditions and mitochondrial function.

Patients with acute liver failure or acute-on-chronic liver failure are classically viewed as having decreased citrate metabolism. However, it has been recently suggested that these patients could instead process citrate, while the markers of liver function are poor predictors of the risk of citrate accumulation [21,22,23].

Regarding the reduction of mitochondrial metabolism, it is present in circulatory shock with decreased oxygen delivery and decreased Krebs cycle activity. This condition can also happen with intoxication with metformin, cyclosporine, paracetamol, trichloroethylene, or propofol. All these clinical conditions are typically associated with elevated serum lactate levels, whose threshold above which RCA should not be used remains to be determined [24].

The use of a CKRT-RCA management protocol therefore becomes essential to promptly identify and treat complications related to citrate use. Among these complications, alkalosis and acidosis can be treated by varying the citrate load, while the onset of citrate accumulation has to be managed in relation to the patient’s clinical conditions and risk of developing clinically significant hypocalcemia.

Literature reports different protocols for the management of CKRT treatments in RCA [25,26,27,28] in addition to those provided by the manufacturers of CKRT monitors.

The aim of this study is to demonstrate the validity and safety of a protocol we have performed regarding the prevention of citrate accumulation in patients treated with CVVHDF-RCA. To this end, we have looked at the complications recorded during the application of our specific monitoring protocol of CKRT treatments in RCA, considering also the dynamic time windows for the total calcium and ionized calcium controls in order to be able to detect an initial deviation of these parameters from the physiological ranges.

Our protocol applied during the observation period of the study, which reached more than 55,000 h of CKRT treatments in RCA, proved to be safe and effective for the early detection of the observed complications.

## 2. Materials and Methods

In this study, we analyzed all CKRT prescriptions provided by the Nephrologists of the Nephrology Unit of the Department of Medicine of the University of Padua from December 2020 to April 2022 in the 6 Intensive Care Units (ICUs). CKRTs were prescribed according to local clinical practice and the use of biomarkers for AKI [29,30]. The main indications for CKRT treatment were urea > 35 mmol/L, potassium > 6.0 mEq/L, severe acidosis with pH < 7.10, creatinine > 400 mmol/L, refractory pulmonary edema, hypernatremia > 160 mmol/L, anuria for more than 12 h [31], fluid overload in COVID-19 patients poorly responsive to i.v. diuretic treatment, AKI, and sepsis-induced AKI in COVID-19 patients [32]. These CKRT prescriptions are routinely recorded in the informatics system with the lab tests, while clinical parameters and CKRT complications are obtained from the patient’s electronic health records.

The study was conducted in accordance with the Declaration of Helsinki. Ethical review and approval were waived for this study as required by the retrospective clinical investigation; the local ethics committee was informed (protocol number 46872). Data on CKRT prescriptions, lab tests, clinical conditions, and complications of citrate use in CKRT were collected anonymously and pooled for all adult patients. The anonymization process prevented any possible transmission of sensitive data, preserving subject privacy. The collected data describe the CKRT complications of local clinical practice and are the result of an unfunded spontaneous single-center observational study. The CKRT treatments were performed with the use of a Prismax or Prismaflex monitor (Baxter, Deerfield, IL, USA) with ST-150, ST-100, oXiris, or Septex kits (Baxter, Deerfield, IL, USA), depending on the clinical indications. The hemodiafiltration solutions used in RCA were: PrismaCitrate 18/0 for the regional anticoagulation with citrate of 18 mmol/L; PrismaCal B22 for the dialysate; Prismasol 2, Prismasol 4, or Phoxilium (Baxter, Deerfield, IL, USA) for the reinfusion in post-dilution. All CKRT-RCAs were provided by CVVHDF-RCA. For the use of the Septex kit, a solution of citrate 4% (136 mmol/L of citrate) (Citrasol 4%, BBraun, Melsungen, Germany) was used instead of PrismaCitrate 18/0 in CVVHD-RCA due to the high cut-off of the filter membrane that avoids the use of convection. All CKRTs (CVVVH, CVVHD, CVVHDF) with heparin or without anticoagulation were performed by the same monitors with the same filters with Prismasol 2, Prismasol 4, or Phoxilium as hemodiafiltration fluids. The extracorporeal blood circulation was performed by a 12 G dual-lumen CVC for hemodialysis placed in a jugular or femoral vein.

According to the K-DIGO guideline, the effluent dose [33] was calculated on the basis of the patient’s weight in order to reach an administered dose of at least 20–25 mL/Kg/h considering the downtimes in the specific patient’s care setting. The prescribed dose in CVVHDF-RCA was corrected for the dilution factor and divided into the three different flow components: Pre-Blood Pump (PBP) as pre-dilution infusion (effluent dose in convection), dialysate (Q_d_) (effluent dose in diffusion), and post-dilution infusion (Q_r-post_) (effluent dose in convection); all flows were expressed in mL/h, while the blood flow was in mL/min [34]. The ratio of blood flow (mL/min) to PBP and dialysate (mL/h) was kept at 1:10:10 to maintain a theoretical removal of approximately 50% of infused citrate. The amount of Q_r-post_ was used to ensure the achievement of the required effluent dose without the need to use high blood and PBP flows corresponding to a higher citrate load for the patient, especially in patients with body weights above 90 kg. In CKRTs without citrate, the effluent dose was calculated based on the patient’s weight in order to reach the effluent dose as per the guideline [33], and the total dose was divided into the three flows according to the nephrologist’s prescription without using a standard protocol.

All patients treated with CVVHDF-RCA had transaminases below 250 IU/L and lactic acid below 8 mmol/L at the moment of prescription.

A specific protocol to manage blood citrate and calcium compensation was used in all patients to provide an effective circuit of regional anticoagulation, reaching a physiological level of ionized calcium in the patient over time (Table 1). The protocol is aimed at detecting an inadequate systemic calcium level requiring correction by the evaluation of calcium compensation and an inadequate ionized calcium level in the circuit requiring blood citrate adjustment. The protocol can identify an initial citrate accumulation in the patient as valued by the Ca_Tot_/Ca^2+^ ratio and the continuous increase in the calcium compensation over time without ionized calcium correction. Table 1 shows the time intervals for performing calcium checks during the treatment according to the dynamic calcium trend.

For each determination of ionized calcium, the protocol also provides for the control of blood Na^+^, K^+^, and Cl^−^, bicarbonate levels, pCO_2_, and pH. Bicarbonate levels, pCO_2_, and pH allow for the diagnosis of a state of metabolic acidosis or alkalosis, requiring changes in the patient’s citrate load and determining changes in bicarbonate levels after the citrate metabolism.

The protocol envisages considering the Ca_Tot_/Ca^2+^ ratio between 2.40 and 2.50 as indicative of possible citrate accumulation, while a value greater than 2.50 as indicative of citrate accumulation [35]. Systemic ionized calcium values are considered in the normal range between 1.00 and 1.20 mmol/L, while post-filter ionized calcium values are between 0.25 and 0.35 mmol/L.

**Figure 2:**
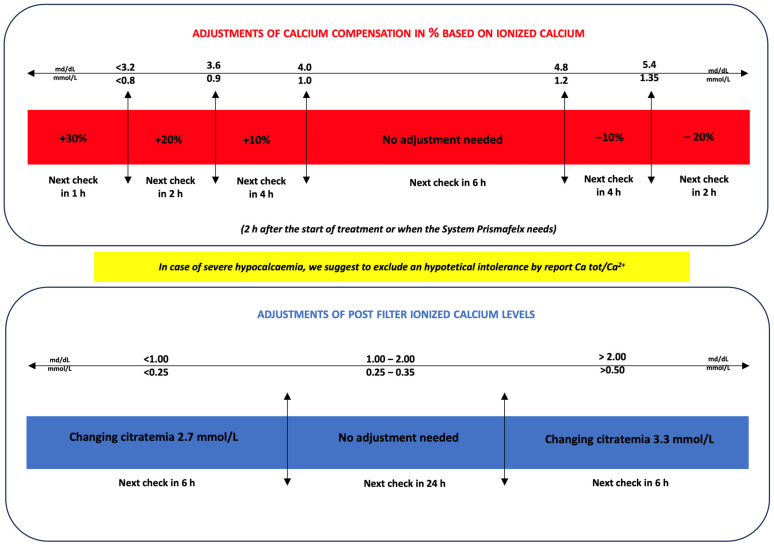
Adjustments of calcium compensation and blood citrate based on systemic ionized calcium and postfilter values, respectively, during the CVVHDF-RCA treatment.

The algorithm, shown in Figure 2, predicts changes in calcium compensation related to systemic ionized calcium levels and changes in blood citrate related to post-filter ionized calcium levels.

Each check of the systemic ionized calcium and post-filter ionized calcium was followed by an evaluation by the algorithm (Figure 2). The standard 6 h check is provided at the steady state only if the systemic and post-filter calcium are in the normal range, while a 2 h check is required if a parameter is changed. These parameters have to be checked every 2 h until 6 h. When the steady state is reached with no changes in blood citrate or calcium compensation, the system can be checked every 6 h. In the case of calcium compensation variation greater than −20% or +30%, a check at 2 and 1 h is required, respectively, and then the system can be checked at 6 h.

**Figure 3:**
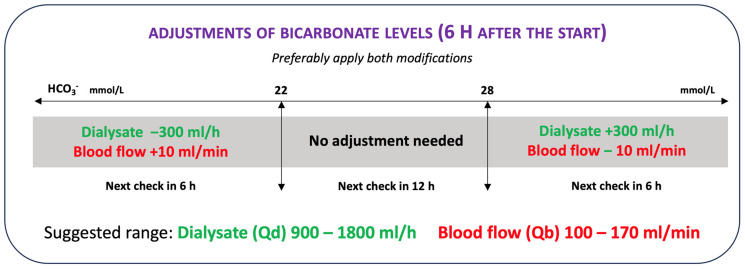
Adjustments of blood flow or dialysate flow to the bicarbonatemia correction.

At the same time, the protocol provides control of pH, PCO_2_, and systemic bicarbonates to detect states of alkalosis or acidosis. According to the algorithm (Figure 3), it is possible to increase or decrease the citrate load in the patient to change the bicarbonate level as required by the acid-base patient’s status. The citrate load can be changed by variations in blood flow, dialysate flow, or blood citrate. An increase in blood citrate or blood flow or a reduction in dialysate flow leads to an increase in the patient’s citrate load and therefore in bicarbonate. Conversely, a reduction in blood citrate or blood flow or an increase in dialysate flow results in a reduction in the patient’s citrate load and therefore in bicarbonate. Any variation in these parameters must always be followed by a check of the ionized systemic calcium and a 2 h post filter check.

Each variation in the parameters of CVVHDF-RCA treatment was recorded in the patient file and certified by a digitally signed medical prescription to ensure data traceability and prescription safety. In cases of hypokalemia and/or hypophosphatemia, the post-dilution hemodiafiltration fluid was replaced with the proper fluid to avoid the infusion of electrolytes for the correction of the disorder [36].

## 3. Results

Among all 374 patients treated with CKRT in the ICUs, 104 received the first prescription for CVVHDF-RCA (Table 2). 30 patients (29%) showed a positive molecular test for SARS-CoV-2, while 15 (14.5%) presented with severe SARS-CoV-2 pneumonia.

A total of 80 patients were males (77.7%) and 24 females (22.3%), with a mean age of 67.0 ± 11.3 years (min. of 19 years and max. of 87 years). The mean weight was 82.0 kg ± 19.6 kg (min. of 30 kg and max. of 130 kg). 26 (25%) out of 104 patients had pre-existing CKD, while 78 (75%) did not have a documented history of CKD.

A total of 52 patients (50%) at the time of the nephrological evaluation presented anuria for more than 12 h, while 26 (25%) presented oliguria for more than 12 h. The remaining patients presented a preserved diuresis with progressive alteration of the renal function. The nephrological consultation was urgent in 68 (65.4%) patients due to their critical condition requiring in 18 (17.3%) the use of vasoactive amines for the maintenance of blood pressure, in 32 (30.7%) mechanical ventilation, in 6 (5.8%) the use of ECMO (ExtraCorporeal Membrane Oxygenation), and in 3 (2.9%) the use of L-VAD (Left Ventricular Assist Device). In 36 patients (34.7%), the consultation was scheduled due to a stable clinical condition. At CKRT prescription, the mean urea values were 26.53 ± 3.29 mmol/L (min. 4.80 mmol/L and max. 59.00 mmol/L), the mean serum creatinine was 321.0 ± 205.2 µmol/L (min. of 43.0 µmol/L and max. of 1191.0 µmol/L), and the mean potassium values were 4.44 ± 0.73 mmol/L (min. 3.00 mmol/L and max. 7.20 mmol/L).

The total number of treatments performed in this patient population was 806, for a mean of 7.75 ± 8 circuits (median of 5 circuits, minimum of 1, and maximum of 34 circuits).

CVVHDF-RCA was used in 104 patients (27.8%) during the study period, with a citrate dose of 3 mmol/L in 88.5%, a dose of 2.5 mmol/L in 8.7% of treatments, and doses of 2 mmol/L and 2.7 mmol/L in only 2.8% of patients. The mean calcium compensation was 99.0 ± 9%, with a minimum value of 55% and a maximum value of 150%. The average dialytic dose prescribed was 37.9 ± 9.3 mL/kg/h, with a minimum of 16.9 and a maximum of 74.3 mL/kg/h.

The mean Ca_Tot_/Ca^2+^ ratio was 2.08 ± 0.18 (min. 1.89, max. 2.45), the systemic ionized calcium was 1.14 ± 0.06 mmol/L (min. 1.05, max. 1.25), the total calcium was 2.29 ± 0.12 mmol/L (min. 2.11, max. 2.45), lactate was 2.47 ± 1.48 mmol/L (min. 0.80, max. 7.70), and bicarbonates were 22.96 ± 3.58 mmol/L (min. 16.3, max. 33.4) mmol/L.

**Figure 4:**
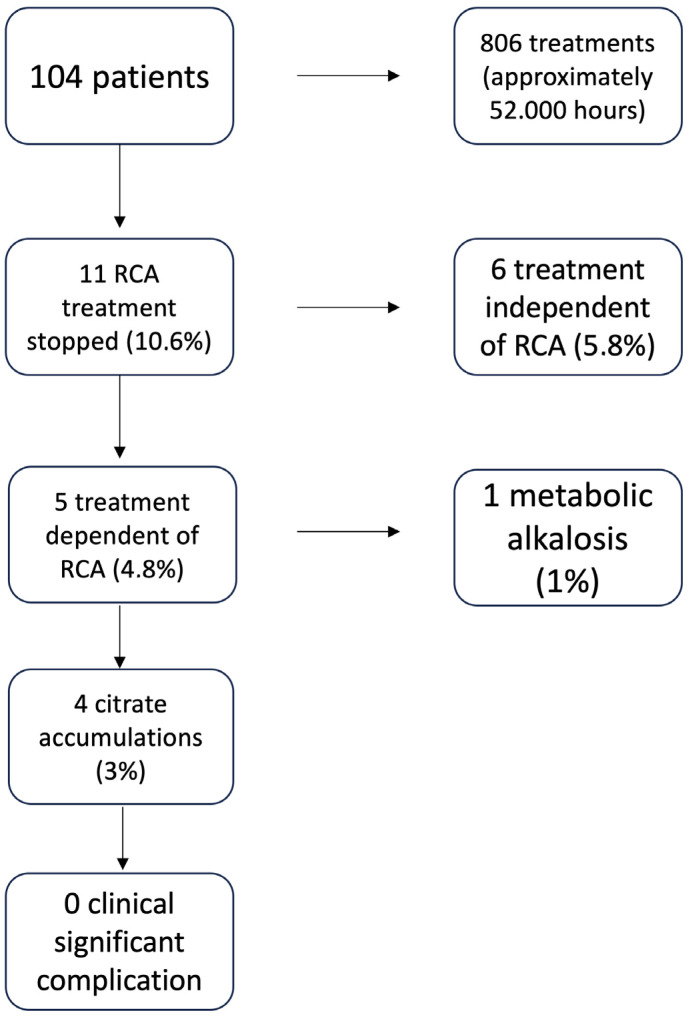
Discontinuation of the treatment CVVHDF-RCA due to the complications of citrate use.

In 11 patients (10.6%), it was necessary to discontinue the treatment with sodium citrate (Figure 4). In all these cases, CKRT without anticoagulation was performed. In detail, 4 treatments were discontinued due to a suspicion of citrate accumulation as noted by a Ca_Tot_/Ca^2+^ ratio between 2.40 and 2.50, 1 was discontinued due to the development of metabolic alkalosis (pH 7.55), 1 was suspended to allow infusion of higher doses of bicarbonate via a different CKRT procedure due to a state of severe metabolic acidosis that could not be corrected by the infusion of high doses of citrate, 4 were suspended due to the increase of hepatocytolysis indexes, and 1 patient was preemptively discontinued from CVVHDF-RCA t following a massive bleeding with lactate elevation up to 7 mmol/L after abdominal surgery, even in the absence of biochemical alterations that could hypothesize an initial accumulation of citrate. None of the patients had citrate toxicity as indicated by a Ca_Tot_/Ca^2+^ greater than 2.50.

It is interesting to note that the 104 patients used a total of 806 circuits for more than 52,000 h of citrate treatment. Using a Ca_Tot_/Ca^2+^ ratio of 2.40 to implement changes from 24 to 12 h total calcium checks allowed treatments to be discontinued for suspected citrate accumulation before the ratio exceeded 2.50, which is known in the literature as the cut-off for clinical citrate accumulation [35]. Due to the use of our protocol, 11 out of 104 patients under CVVHD-RCA were discontinued: 6 were discontinued for a citrate independent cause (5.8% of the total treated patients), 5 were discontinued for a citrate dependent cause (4.8% of the total treated patients), 1 due to metabolic alkalosis (1% of the treated patients), and 4 due to a Ca_Tot_/Ca^2+^ ratio between 2.40 and 2.50, not yet indicative of clinical citrate accumulation but likely indicative of possible development of its accumulation; at the time of discontinuation, no clinical signs of citrate intoxication were detected.

## 4. Discussion

RCA is now recommended as an anticoagulation choice for CKRT in patients without contraindications [37].

The most feared and potentially lethal complication of RCA is citrate accumulation, and its diagnosis remains relatively complex in the absence of citrate blood level monitoring, a specific protocol for the patient’s total and ionized calcium monitoring, and trained personnel for the treatment administration [38]. However, when a strict protocol is followed, this complication is rare.

In clinical practice, citrate accumulation has to be distinguished from other conditions resulting in acid-base abnormalities during RCA, such as citrate net overload or insufficient citrate delivery, that can determine alkalosis or acidosis, respectively [39].

We performed a single-center observational study on 374 patients with AKI under CKRT; 104 patients (27.8%) received CVVHDF-RCA treatment. On these latter, we have applied a CVVHDF-RCA management protocol we have specifically designed, which includes the reduction of the Ca_Tot_/Ca^2+^ ratio cut-off from 2.50 to 2.40 for earlier detection of citrate accumulation. The intervals for the systemic ionized calcium checks were maintained every 6 h in the case of the parameters being in the physiological range, while they were taken at 2 h for 3 consecutive times after the variation of calcium compensation if systemic ionized calcium was out of range. The same applies for the variation of blood citrate for the post-filter ionized calcium [40] out of range. When the Ca_Tot_/Ca^2+^ ratio is in the range 2.40–2.50, the protocol requires total calcium checks every 12 h in order to better estimate the Ca_Tot_/Ca^2+^ ratio variation during the 12-h period. The increased number of checks of the ionized calcium acquires more evaluations of the acid-base status by promptly detecting changes in blood bicarbonate requiring changes in blood flow, dialysate flow, or blood citrate.

The increase in the number of checks and the reduction of the Ca_Tot_/Ca^2+^ ratio made it possible to obtain a more restrictive policy on the maintenance of the treatment and the need for variations of the treatment parameters to ensure greater safety.

In our patients, 11 (10.6%) discontinued the treatment with sodium citrate. In particular, 4 patients (3%) were discontinued due to a suspicion of citrate accumulation as noted by a Ca_Tot_/Ca^2+^ ratio between 2.40 and 2.50. In this specific case, the reduction of the Ca_Tot_/Ca^2+^ ratio cut-off determined to discontinue the treatment before a clinically significant accumulation of citrate could occur, avoiding exposing the patient to clinical risk. Therefore, our protocol allowed us to identify the most critical patients who could have presented with citrate intoxication in the event of a sudden decrease in mitochondrial metabolism due to the worsening of a clinical condition such as a reduction in organ perfusion, hypovolemic shock, or heart failure. The reduction of the cut-off of the Ca_Tot_/Ca^2+^ ratio allows the opening of a window of intensive patient observation to be able to discontinue the treatment before it leads to citrate accumulation.

In 1 patient (1%), the CVVHDF-RCA was discontinued due to the development of metabolic alkalosis (pH 7.55, pCO_2_ 38 mmHg, HCO_3_^−^ 33 mmol/L) that has been treated with a CKRT without anticoagulation, which avoided the need to reduce blood citrate, blood flow, or increase dialysate in relation to the patient’s clinical conditions.

In 1 patient (1%) the CVVHDF-RCA was discontinued to be able to infuse bicarbonate via a different CKRT given the patient’s severe metabolic acidosis that cannot be corrected by the infusion of high doses of citrate for the possible development of electrolyte abnormalities and alterations of the effluent dose following the high blood flows or reductions of the dialysate flow.

In 4 patients (3.8%), the treatments were suspended due to the elevation of hepatocytolysis indices following the worsening of the patients clinical conditions due to organ hypoperfusion, and, finally, 1 patient (1%) was preemptively discontinued from CVVHDF-RCA following massive bleeding with lactate elevation up to 7 mmol/L after surgery, even in the absence of biochemical alterations that could hypothesize the initial accumulation of citrate. In these latter 4 patients, the increase in transaminases above the value of 200 IU/L and, in 1 more patient, the increase in lactic acid were the causes of the preventive suspension of the CVVHDF-RCA, even in the absence of a Ca_Tot_/Ca^2+^ ratio and ionized systemic calcium alterations. The preventive discontinuation of the treatment was induced by a restrictive policy to prevent an accumulation of citrate in the event of a decreased mitochondrial metabolism due to intercurrent clinical problems or patient destabilization.

None of the 11 patients showed citrate toxicity as indicated by a Ca_Tot_/Ca^2+^ greater than 2.50.

The application of our protocol was able to detect 11 patients at risk for citrate complications: in 6 patients, the CVVHDF-RCA discontinuation was due to a cause independent from citrate use, while in the other 5 patients, 4 presented a Ca_Tot_/Ca^2+^ ratio in the range of 2.40–2.50, indicating a possible risk for the development of citrate accumulation.

Our protocol introduces the repetition of the total calcium check every 12 h when the Ca_Tot_/Ca^2+^ ratio exceeds 2.40. This allows us to continue observing the patient using a total calcium value that reflects the real condition of the patient by revealing a potential citrate accumulation. The possibility of having more total calcium measurements in 24 h increases the possibility of identifying the trend for a worsening of mitochondrial metabolism and an increase in CCC.

Our data demonstrated that this protocol prevents the development of citrate accumulation, classically defined in the literature as a Ca_Tot_/Ca^2+^ ratio higher than 2.5 [39]. Taking into consideration the cut-off of 2.4, the number of patients with a possible accumulation of citrate was 4 (3.8%) of our patients.

## 5. Conclusions

The use of RCA is rapidly increasing. The hardware and software complexity of RCA monitoring requires trained personnel to appropriately manage the treatment and identify the patient with citrate accumulation. In addition, diverse RCA complications due to citrate deficiency or excessive infusion with respect to the patient’s acid-base balance requirements have to be considered. RCA protocols should aim to minimize the amount of net citrate load delivered to the patient. In the presence of acid-base derangement during RCA, clinicians should be able to differentiate a benign citrate net overload (alkalosis) from a life-threatening accumulation, which should prompt therapy tuning and early termination. The distinction between these two entities is crucial to enabling adequate patient surveillance while receiving RCA. In the patients of our study, the application of a strict protocol allowed us to identify conditions at risk for clinically relevant accumulation of citrate. The results obtained in this study, where our protocol has been used for treatments with RCA, which reached more than 55,000 h, confirm that the protocol proved to be safe and effective for the early detection of the observed complications.

## Figures and Tables

**Table 1:** Protocol for the control of total and ionized calcium during the CVVHDF-RCA treatment.

Time from the Beginning of the CVVHDF-RCA	Total Calcium in the Patient	Ionized Calcium in the Patient	Ionized Calcium Post-Filter	Ca_Tot_/Ca^2+^
0	X	X		
30′		X	X	
3 h		X	X	
6 h		X	X	X
Every 6 h		X	X	
Every 24 h	X			X
(*) Every 12 h	X			X

In the event of a change in blood citrate or calcium compensation, a check is scheduled every 2 h and then every 6 h as per protocol. In the case of a Ca_Tot_/Ca^2+^ value in the range 2.40–2.50 (*), the protocol requires checking the total calcium every 12 h instead of every 24 h, as in a stable clinical condition. In the case of Ca_Tot_/Ca^2+^ > 2.50, the treatment has to be immediately discontinued on suspicion of clinical citrate accumulation.

**Table 2:** Patients’ characteristics.

104 Patients 80 Males (77.7%) 24 Females (22.3%)	Mean	Standard Deviation	Minimun	Maximum
Age (years)	67.0	11.3	19	87
Weight (Kg)	82.0	19.6	30	130
Urea (mmol/L)	26.53	13.29	4.80	59.00
Creatinine (µmol/L)	321.0	205.2	43.0	1191.0
Potassium (mmol/L)	4.44	0.73	3.00	7.20
Q_b_ (mL/min)	123.6	80	80	200
PBP (mL/h)	1203.6	212.7	800	1800
Q_d_ (mL/h)	1190.6	223.7	800	2000
Q_r_ (mL/h)	597.1	235.0	100	1600
Effluent dose (mL/Kg/h)	37.9	9.3	16.9	74.3
Albumin (g/L)	26.5	6.1	21	50
Mg (mmol/L)	0.89	0.129	0.67	1.59

## Data Availability

Not applicable.
